# The development of a globally acceptable national model for occupational hygiene in Turkey: a modified Delphi study

**DOI:** 10.1186/s12992-019-0480-z

**Published:** 2019-06-13

**Authors:** Sibel Kiran, Alp Ergor, Ceyda Sahan, Esra Emerce, Sergio Luzzi, Yucel Demiral

**Affiliations:** 1 Hacettepe University, Institute of Public Health, Department of Occupational Health and Safety, Sihhiye-Ankara, Turkey; 2 Dokuz Eylul University, Department of Public Health, Izmir, Turkey; 3 Dokuz Eylul University, Department of Public Health, Department of Occupational Medicine, Izmir, Turkey; 4 Gazi University, Pharmacy Faculty, Department of Toxicology, Ankara, Turkey; 5 University of Florence, National Secretary of AIDII (Italian Industrial Hygienists Association), Florence, Italy; 6 Dokuz Eylul University, Department of Public Health, Department of Occupational Medicine, Izmir, Turkey

**Keywords:** Occupational health, Occupational hygiene, Industrial hygiene, Professional practice, Policy development, Modified Delphi

## Abstract

**Background:**

Although various organizations working in developed countries established the standards and approaches used in occupational hygiene, occupational hygiene professional interests and needs continue to develop in a global context. There is thus an urgent need for expanded occupational hygiene models. For successful field implementation, these models should be based on several sets of criteria, including those related to international standards, various national requirements, and multidisciplinary approaches. This is particularly important for countries in which no occupational hygiene model has been developed. This study thus examined the consensus on occupational hygiene standards among stakeholders in Turkey regarding the development of a national model. A modified Delphi study was conducted among key occupational health experts in Turkey who could aid in the relevant implementation, policy-making, and educational processes for such a model. Participants were selected from various governmental institutions, non-governmental organizations, trade unions, universities, and occupational health practices.

**Results:**

The first-round findings were obtained from open-ended questions. The results revealed several requirements, including the adoption of an international hygiene definition, the official recognition of professional and practical areas in Turkish occupational hygiene, hygienist training methods, priorities, and competent institutions. Second-round findings indicated a consensus rate of over 80% regarding the need for implementation standards, training and education standards, requirements and priorities, and competent institutions for professionals working in the field of occupational hygiene. A third-round and SWOT analysis was also conducted among the group to confirm the consensus issues.

**Conclusions:**

The search for solutions and developmental expectations increases when awareness of internationalization and the need for common global standards increase. This developmental process may provide the basis for an appropriate model in developing countries.

## Background

The need for standardized qualifications, training, and career development programs will determine the global professional perspective for occupational hygiene in the developing world [1–5].

To use a phrase from the International Labour Organization (ILO), global developments in the “world of working life” create new challenges for all professions. This does not only occur in developing and emerging economies; it is also relevant in established workplaces in the developed world [6].

In the field of occupational hygiene, it is important to provide a standardized, mutual approach in the local context while also exchanging knowledge on a global scale [7, 8]. The ILO defines occupational hygiene as the science by which professional practices anticipate, recognize, evaluate, and control hazards in or from the workplace so that “different ways of definitions all have same meaning for the fundamental goal of protecting and promoting the health and well-being of workers, as well as general environment, and preventive actions in the workplace” [9]. Beyond the need for general definitions, requirements, and standards for occupational/industrial hygienists, there should also be similar approaches to providing service. Professional occupational hygienists can also support health and well-being through both multidisciplinary and interdisciplinary approaches. This is not only useful and efficacious for occupational hygienists, but also other occupational health professionals. In addition, “the professional area of occupational hygiene practice should be included in the political agenda as a business model” to plan for future standards in the field [4, 10]. From this perspective, global action, multi-professional approaches, and cross-disciplinary integration are necessary to achieve the essential aim of establishing occupational hygiene as a professional practice [1].

Such a practice requires different professions and technical expertise when attempting to reach a consensus for the creation of a standardized perspective. This is necessary to manage related policy issues, including local challenges in emerging economies [4, 11]. Here, there is a relevant example from the perspective of both the World Health Organization (WHO) and the ILO regarding training practices in India [12]. That is, both organizations stated the need for international collaboration to improve standards but did not mention an integrated model for establishing stakeholders as part of a global professional workforce in developing countries [10, 12–14].

Future policies will be affected by professional needs and perspectives according to current perception, especially in countries with a “complex world [in the working] environment” (e.g., Turkey). The occupational hygiene field requires a consensual understanding of the practical and applied needs. This would aid in the creation of an improved model for understanding global needs in countries with similar conditions [15].

The occupational hygiene field is broad in scope and contextually specific. Here, individual workplace settings require influential roles that depend on the background. They also require similar recognition. Occupational health has evolved into a more holistic, public health-oriented model that requires engagement with a wide network of stakeholders [16]. Collaboration and cooperation are thus necessary from all stakeholders for the improved implementation and development of the occupational hygiene field. This is a requirement for the field to evolve in an occupational health context.

The International Organizations of Hygiene Associations (IOHA) described the various roles within the occupational hygiene field (i.e., certified practitioners, professional occupational hygienists, educators, and managers/administrators or consultants, depending on the position). The IOHA highlighted that the functions within these roles would expand due to the economic, sociopolitical, and technological demands of specific industries, sectors, and businesses. Several occupational hygiene associations (e.g., the Occupational Hygiene Training Association, British Occupational Hygiene Society, American Industrial Hygiene Association, Australian Institute of Occupational Hygienists, and Associazione Italiana Degli Igienisti Industriali) have established educational programs and competencies in the field of occupational hygiene. These programs were developed according to specific certification schemes (some are available on the following websites: https://ioha.net/, http://www.ohlearning.com). This has led to the development of specific definitions for occupational hygiene practices and roles according to each country’s professional members. These terms are based on different levels of practice or profession (e.g., those that apply to occupational hygienists, registered hygiene practitioners, experts, consultants, and technicians). There is also a classical definition and several descriptions, but there is a need for a clear demonstration of new roles based on recent occurrences in developing countries [10, 17, 18].

Although a broad range of practical applications and technical duties can be found among various occupational health and safety professions, Turkey has no professional definition for occupational hygienists, nor does it have an integrated model for occupational hygiene [19, 20]. However, there are more than 100,000 occupational safety experts and 3300 company/factory physicians working in Turkey [21].

The EU funded project Occupational Health and Safety Networking in Eastern Europe (OSH EAST NET) project that was implemented between 2008 and 2010, as part of the IPA funding line by a consortium coordinated by the Italian Industrial Hygienists Association (AIDII). The program included certain Balkan countries and Turkey [22]. The project results revealed that occupational hygiene was not described as a professional post in Turkey, but the educational and training frameworks of the project were provided to project managers and Turkish representatives. By the collected data and results of joint meetings derived that Turkish Occupational Health and Safety system might have been ready for productive and effective improvements inspired by the principles of the occupational hygiene approach. Subsequently, a training scheme was developed in response to the OSH EAST NET project, and since 2011, a structured series of courses titled the “OSHNET School” have been implemented in various parts of the country. These courses provide both full fundamental training and specific thematic modules in the field of occupational hygiene [22].

This study was conducted in collaboration with the OSHNET School. Research proceeded according to a program and prioritization approach based on the need to determine the requirements for adopting the professional areas and concepts for occupational hygiene in Turkey while also developing an international approach for occupational hygiene concepts and applications [22].

We investigated examples from the U.S. National Institute for Occupational Safety and Health’s hygiene capacity structure in developing countries to contribute to the international literature for the development of an occupational hygiene model. This was done to seek a consensus between occupational health parties toward the implementation of national requirements in the scope of international OH training schemes [1, 18]. The USA model is not fully suitable for developing countries due to having different actors and complex parties. It is necessary to develop new models by considering different infrastructures and human resources in transition countries such as Turkey.

This study aimed to provide examples for planning and sharing a common perspective for the establishment of a consensus for providing professional occupational hygiene services in Turkey and other countries with similar requirements.

The final goal was to collect the opinions of key experts working in occupational health and safety regarding occupational hygiene. This was completed to provide information for a framework for use among stakeholders in clarifying the roles and responsibilities of occupational hygienists working in Turkey.

## Results

### Round 1: Evaluating the open-ended questions

With a suitable distribution from the “tripartite” structure of occupational health implementation logic, an open-ended structured call was made to a group of 32 people out of 58. Respondents were field experts and members of government and education institutions (the accessibility rate was 71.8%). Table 1 summarizes the group distributions over three rounds (the first round included participants from government institutions [39.1%], NGOs and trade unions [39.1%], and universities [21.8%]).

**Table 1:** Basic participant characteristics for each of the three rounds

Features	Round 1 [*n* = 23]		Round 2 [*n* = 47]		Round 3 [*n* = 41]	
	n	%	n	%	n	%
Sex						
Female	7	30.4	16	34.0	11	26.8
Male	16	69.6	31	66.0	30	73.2
Institution*						
Government	9	39.1	7	14.9	14	34.1
NGO, or trade union	9	39.1	5	10.6	20	48.8
University	5	21.8	11	23.4	7	17.1
Occupational health practice	–	–	24	51.1	–	–

*Government institutions included the Ministry of Labor and Social Security, Ministry of Health, Ministry of National Defense, Ministry of National Education, Provincial Health Directorate, and the Provincial Directorate of National Education. NGOs and trade unions included both associations and unions. Universities included direct university employees. Occupational health practices included occupational physicians and occupational safety specialists working in the private sector

Following the participant grouping during the first round of open-ended questions, the second round included participants from government agencies (14.9%), NGOs and trade unions (10.6%), universities (23.4%), and occupational health professionals (51.1%).

The first-round findings illustrated the requirements for adopting an international hygiene definition, agreeing about the definition, occupational hygiene practices in Turkey, officially recognizing the professional and practical areas of occupational hygiene, training occupational hygienists, priorities, competent institutions and corporate functions, and the steps to be taken.

### Round 2: Level of consensus

A total of 73.9% of the first-round participants also participated in the second round. We analyzed the responses to the second questions by averaging the agreement level to produce a mean score for each aspect and frequency of the level of agreement for the whole group within the expressed opinion items.

We then analyzed responses to the second questions by averaging the agreement level to produce a mean score for each aspect and frequency of the level of agreement for the whole group within the given aspects.

In this Delphi, we decided that a valid consensus was achieved through a participation rate of over 80% regarding the suggestions in eight main priority areas for the development of occupational hygiene.

### Round 3: Priorities

The above processes and related results were shared during the third round. A forum, workgroup, and SWOT analysis were also conducted.

The information derived during the group sessions was received before the SWOT analysis on the steps to be taken. This was presented to all parties when sharing the report. These steps were grouped as occupational hygiene practice standards, training standards services, and the functions of competent people and institutions. Table 2 illustrates the consensus and participation levels for the prioritization scores regarding the key requirements and steps that should contribute to the final model. The group session was concluded with a SWOT analysis.

**Table 2:** Statements contributing to the model development process (participants reached the highest and lowest consensuses)

Statements	Consensus levels	
	% of agreement	Mean ± SD*
Implementation standards for occupational hygiene in Turkey		
*Highest agreement statements*		
Occupational hygiene practices should not be limited to measurements and controls; preventions should also be included.	97.9	6.7 ± 0.8
Occupational hygiene implementation standards should be determined.	93.6	6.6 ± 1.0
*Lowest agreement statements*		
Occupational hygiene practices should be carried out by occupational physicians.	34.1	3.7 ± 1.9
Occupational hygiene practices should be carried out by occupational safety experts.	48.9	4.2 ± 1.8
Training and education standards for occupational hygiene in Turkey		
*Highest agreement statements*		
Occupational hygiene training and education standards should be determined.	93.6	6.6 ± 1.1
Competencies in occupational hygiene issues for occupational safety experts, occupational physicians, and labor inspectors should be increased.	94.7	6.5 ± 0.9
Standards and accredited training/education programs should be required in occupational hygiene.	93.6	6.5 ± 1.1
*Lowest agreement statement*		
The field of occupational hygiene should be developed by risk-specific branching.	87.2	5.7 ± 1.2
Requirements and priorities for occupational hygiene in Turkey		
*Highest agreement statements*		
Awareness of occupational hygiene should be promoted.	95.8	6.5 ± 1.1
Occupational hygiene training should be disseminated according to nationwide authorizations and standards.	91.5	6.4 ± 1.3
*Lowest agreement statement*		
Occupational hygiene should be defined as a profession, and its education/competencies should be determined.	85.0	6.0 ± 1.8
The functionality of competent institutions in Turkey regarding occupational hygiene/hygienists		
*Highest agreement statements*		
Occupational hygiene training and research structures should be established at universities.	93.7	6.5 ± 1.1
The central and regional laboratories at the Institute of Research and Development of Occupational Health and Safety (ISGUM) should be strengthened. Reference laboratories should also be established.	93.7	6.5 ± 1.2
*Lowest agreement statement*		
As a competent organization, MoLSS should establish related training programs and practices through inter-institutional cooperation.	93.7	6.3 ± 1.1
* Rated over 7.		

On the topic of “implementation standards for occupational hygiene in Turkey”; “the existence of authorized laboratories and the relevance of the Ministry and the professional organizations to the issue” were found to be strengths, while “specialization and lack of competencies” were the main weaknesses. SWOT analyzes for “training and education standards for occupational hygiene in Turkey” showed the strength as “the society (TROHA) can work together with the Ministry”, on the other hand “challenges in requirements of multidisciplinary working” was the weakness. The 3rd group has analyzed the subject entitled “requirements and priorities for occupational hygiene in Turkey” and they expressed that “presence of the society and presence of good practicing examples” were the strengths while “absence of occupational hygiene/hygienist definition in the country” was the weakness. The last group worked on “the functionality of competent institutions in Turkey regarding occupational hygiene/hygienists”. While “effective and inveterate structure of the Ministry of Labor and Social Security and having an international relationship of the TROHA” were the strengths, the absence of the standards on education and profession in the area of occupational hygiene in Turkey was the weakness. The opportunity has been found to be having a new society about occupational hygiene such as TROHA. The main threat was changing the system and decision-makers in authorized institutions.

The highest level of agreement (a 97.9% consensus rate) on implementation standards for occupational hygiene in Turkey involved the focus of occupational hygiene practice. The lowest level of agreement about the implementation stage involved the necessary background characteristics for an occupational hygiene practitioner. Here, the consensus levels were 34.1% for a physician background and 48.9% for a safety expert background. There was an 87.2% consensus rate about the need for training and educational standards and an 85.0% consensus rate for the need to recognize occupational hygiene as a profession in Turkey (Table 2).

When looking at the functionality of competent institutions in Turkey, there was a 93.7% consensus about the coordination between universities, the Institute of Research and Development of Occupational Health and Safety, and MoLSS.

## Discussion

This study revealed a high-level consensus about the need for definition and practice standards in Turkey, for future developments between the parties in the fields of sustainability, clarification, professional background, and the mixed applications and practical issues in a transitional period regarding occupational hygiene. According to the ratification of ILO Convention 161, Turkey should implement new regulations about occupational hygiene and occupational health and safety. There is defined need of qualified human resources in OHS services among some of ICOH member countries [6, 21, 23]. In the scope of ILO Convention the OHS professionals from different backgrounds may work in the field of occupational hygiene especially in transition countries where the specified human resources are scarce. This would provide the opportunity to reach international quality standards including ISO 31000 and ISO 45001 (www.iso.org).

The findings indicated a consensus on Turkey’s occupational hygiene priorities and requirements. Here, the evidence indicated that the process was shaped through institutional roles that created a common awareness and established political and relevant legal arrangements in addition to stakeholder cooperation, which also played a role in the process. There was no consensus about the appropriate professional background for a practicing occupational hygienist. However, there was a consensus that those with physician and engineering backgrounds should be trained during the transitional period in which the new professional definition is established.

While occupational hygiene training and implementation programs are certified by professional associations at different levels and through structured standards, the evidence indicates that cooperation is required. This can advance the field through an official recognition process. There is a consensus on the need to improve existing acquisitions by establishing protections, steering distributed knowledge, ensuring application diversity, and realizing the effectiveness of parties/actors through a formalization process.

There was a high level of agreement about implementation standards and functionalities. Here, there was an overlap regarding the standardization of training and education programs and the need for a professional structure for occupational hygiene, thus indicating the desire for global consistency [24–27]. However, the lowest level of agreement was found regarding the implementation stage when looking at the need for increased awareness. This concerned the necessary background characteristics of occupational hygiene practitioners. There is still no functional definition for use in the knowledge transition process; this may shape the progress of knowledge management in the field [17, 18]. There should be a global focus on the need to determine this aspect for future direction. This should involve cross-disciplinary knowledge and interdisciplinary work in occupational health practice and certification programs.

Finally, there was a clear consensus regarding the most important needs (i.e., mutual/common awareness of institutional roles and integrated policies to produce related legislation).

This study combined a modified Delphi and SWOT workshop group to achieve its results, which should be addressed by integrating occupational hygiene in the global context. Standards have changed based on global conditions and through regional integration involving a holistic perspective. This study revealed the need for an international positioning, a common understanding, and a sustainable professional perspective for the future of occupational hygiene in developing countries with no official professional structures. The distributions of the consensus areas and levels may also serve as a basis for creating methods and approaches to achieve robust global standards for occupational hygiene competencies and practices. A policy impact could be achieved by practicing these standards in occupational health and safety globally [27, 28].

The training framework for the OSHNET School courses was defined and conducted in collaboration with AIDII and certain national agencies. The framework is a complete scheme for occupational hygienist competencies according to the international certification for OH competencies recognized by IOHA. Since 2011, two regular course sessions have been organized each year (in spring and autumn). Approximately 300 professionals from different backgrounds have participated.

In this spirit of cooperation, an agreement was established between the OSHNET School and the Institute for the Certification of Prevention Figures (ICFP) in 2017 for the certification of Turkish experts as qualified occupational hygienists. ICFP has recognized the system of credits assigned by the OSHNET School to those receiving positive scores on their final exams as a valid preparatory education certification and training process. ICFP has obtained accreditation ACCREDIA No. PRS 072 C for the certification of personnel in accordance with EN ISO/IEC 17024 standards for certified industrial (occupational) hygienists.

ICFP ensures that occupational hygienists certified through its procedures possess the knowledge, personal characteristics, and necessary work experience to guarantee professionalism in both public and private institutions at an international level. This is derived through ICFP membership in the most important international agreements for mutual recognition (e.g., EA, IAF, and ILAC). The ICFP certification procedure is also recognized by the National Accreditation Committee, which aims to achieve mutual recognition through certification schemes and collaboration between industrial hygienist associations from 25 countries (approximately 20,000 professionals).

In collaboration with the OSHNET School, the final workshop report was shared on the web with open access. Whether they participated in this Delphi study by sending an expert, all relevant national and international institutions agree on the need for basic steps and results to be referenced in political documents [29].

These results may help eliminate a brick from the wall that divides the old and the new regarding the importance of internationalization and cooperation. This is necessary to produce requirements for practical and professional approaches while implementing occupational hygiene standards. A robust and sustainable system will not be possible while this wall continues to divide international professional expertise approaches in the global context (especially without a standardized approach for developed and developing countries regarding a global professional background for a global workforce). A common understanding and common approaches in occupational health, practical standards, and future research will shape the benefits and functionality of knowledge in the occupational hygiene field. Regarding the mutual understanding on the shortfalls/gaps and strengths of existing relevant models on occupational health and safety or hygiene there might be a need to focus on an international multicentric research in the future. In order to provide comprehensive occupational health, safety and hygiene services there is a need for collaboration between the different professionals working in the OHS. On the other hand, there is also need for clarification of the roles and responsibilities of these professionals. One of the important shortfalls of the existing models, in transferring to another country, is education and training systems of the OHS professionals which is not standard accross these countries. This could be regarded as strengths, on the other hand, since each country needs to develop its own system while following the international prerequsities.

The strength of this study is in addition to obtain qualitative data from individuals who work in occupational hygiene from different institution, with combination of workshop this modified DELPHI study design, allowed face-to-face discussions to the participants. This situation was an important stage in ensuring the consensus for future steps and practice. Conducting a qualitative study was important because it allowed the experts to receive detailed views about the topic and to participate in institutional level. Beside, having a high participation rate (82.8%) might show the enthusiasm of the expert participants. There are some limitations as well; notably the selected participants were not necessarily provided with their institutional point of view. Therefore inviting experts from different institutions could not be generalized to being representative of all these institutions.

## Conclusions

This study’s main findings revealed that the search for solutions and expectations for development increase as awareness of internationalization and global common standards increases. An 80% consensus rate was achieved in all areas regarding the statements on occupational hygiene standards in Turkey. These standards included a common curriculum and international standards for all professionals involved in occupational hygiene practices. The results of the application standards indicated an agreement on the need for reference laboratories and authorizations based on accreditation, configuration, and dissemination requirements. Occupational medicine, occupational safety and occupational hygiene are need to be evaluated holistically and should be mutually coordinated among OHS professionals. All the practical and theoretical requirements have been established through IOHA and EU integrated programs, and this creates a global foundation for occupational hygiene.

## Methods

**Fig. 1:**
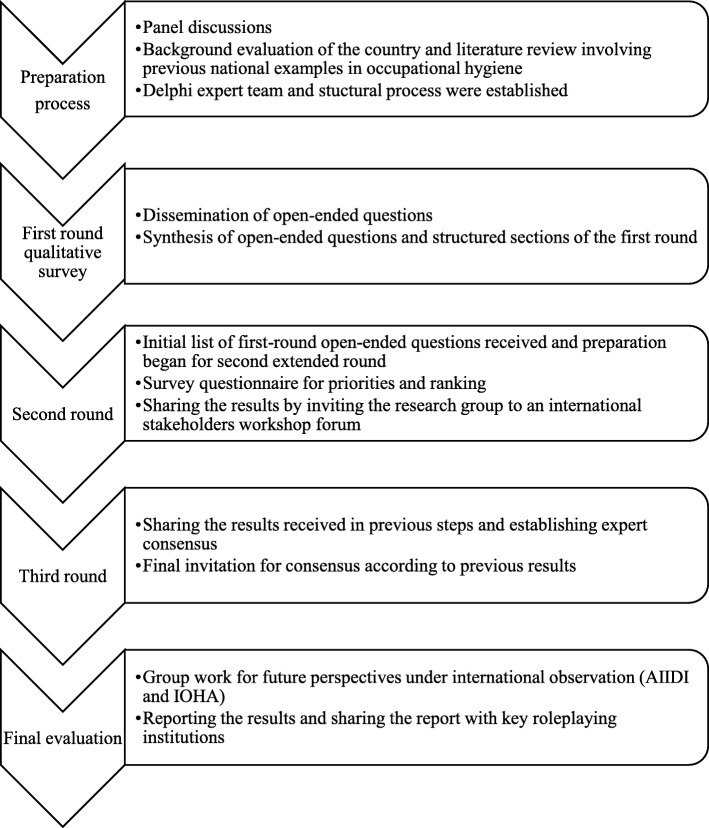
Flow chart illustrating the processes and stages of the modified Delphi study.

This modified Delphi study was conducted among key experts who were stakeholders in the policy-making and training processes for occupational hygiene in Turkey. The study progression is depicted in Fig. 1.

This study’s modified Delphi was conducted with a rigorous, transparent, and systematically solution-based approach according to guidelines suggested in the literature: “Recommendations for the Conducting and Reporting of Delphi Studies (CREDES)” [30].

The researchers did not offer opinions and remained neutral when preparing questions and collecting data. This was done to prevent bias and avoid any potential effects related to the consensus during the Delphi rounds.

We reviewed the relevant literature and country reports on occupational hygiene models and practices. Panel discussions were then organized through electronic meetings and documents were shared via e-mail to consider the status and necessities for building an occupational hygiene model [26, 31–34].

The planning phase consisted of group panel discussions among the research team. The first round questions were prepared according to initial expert opinions in expert panel consensus according to existing literature. First and second round was carried out via an electronic link to open-ended questions regarding topics, suggestions, and requirements.

The panel experts decided on the processes, modifications, applications, variables, and consensus levels. They received opinions from OHS professionals, academicians, and occupational health and safety associations before developing an initial questionnaire and deciding on key experts (KEs) for the survey invitation. The participants of this study were determined by purposive and convenient sampling strategy of qualitative design. Participants were the representatives of those working in the field of occupational hygiene from relevant institutions. The KEs independently provided written opinions during the first stage. The researchers then conducted a progressive panel study to obtain independent opinions on the evaluation themes (i.e., for the invitation, scoping, methodology, and content evaluation rounds). The expert panel conducted its work using a variety of platforms, including face-to-face meetings, electronic opinion platforms, and video interviews. They also requested a consensus on the themes that emerged during the first stage during the second round*.*

The survey questions were sent to a group of KEs consisting of 58 people from 23 institutions in Turkey. These included government entities, employee associations, employer associations, NGOs, and universities. Some relevant examples are the Ministry of Labour and Social Security, the Ministry of Health, the Ministry of National Education, the Provincial Health Directorate, and the Provincial Directorate of National Education. The first round of research was conducted between January and February 2018, while the second round was conducted between March and April 2018, and the third round was conducted in May 2018.

We circulated the questions in Turkish using a survey link via e-mail. Both were piloted in advance to determine ease of use and comprehensibility. We included a participant information sheet at the beginning of each survey and all participants were required to complete a consent agreement before completing the questions. Two reminder e-mails were sent to participants approximately one week after both questionnaires were sent to increase the response rate. The qualitative data were evaluated by the expert panel during its discussion about domains, while the survey’s descriptive results were analyzed using SPSS V.21 [35].

The first round consisted of 14 open-ended questions (Table 3). These were prepared to solicit expert opinions. To prevent bias, the experts were not given information on the answers provided by any other participants. The questions in this round were written and emailed to all participants without any influence from the researchers. We also used neutral statements or questions. We first focused on the definition and context of the term “international occupational hygienist” and asked for information on hygienist training topics. We then focused on topics in the Turkish context suggested by the expert panel and discussed practical global and European standards.

**Table 3:** The first-round questionnaire

1. What do you mean when you say “occupational hygienist”?	
2. What is the current situation in our country regarding occupational hygiene training?	
3. What do you think about the current situation in our country regarding occupational hygiene services?	
4. What do you think about defining occupational hygienist as a professional application area in Turkey?	
5. Does your institution work in the occupational hygienist professional area in Turkey? If so, in what way?	
6. Have institutional policies and strategies been defined for occupational hygienist as a professional area in Turkey?	
7. What is the institutional tendency for occupational hygienist as a professional area in Turkey?	
8. What are the terms and requirements for becoming an occupational hygienist in Turkey?	
9. What training is available for occupational hygienists in Turkey?	
10. What is the status of occupational hygienist applications in Turkey?	
11. What are the setbacks for creating, developing, and implementing a training program for occupational hygienists in Turkey?	
12. What should be done to develop and establish training programs and practices for occupational hygienists in Turkey?	
13. What are the critical approaches that parties require for sustaining advanced occupational hygienist standards in occupational health and safety?	
14. What do you think your institution can do to help develop occupational hygienist as a profession?	

Concepts and definitions involving international occupational hygiene were then reviewed. We examined priority topics in international training, the Turkish topics suggested by the research team, and Global and European topics, and applications, and standards for occupational hygienists [1, 2, 26, 36–38]*.*

The scope of the second round was determined after evaluating and classifying the open-ended information obtained during the first round. We then produced a second-round questions. This included eight main expressed items (Table 4). These items revealed important topical areas for occupational hygiene. We also asked each participant to list priority topics and invited them to help establish a consensus.

**Table 4:** Second-round dimensions

1. Expressions of the approach to adopt a definition for international occupational hygiene	
2. Expressions related to the requirement of an agreement on the definition of occupational hygiene	
3. Expressions that describe occupational hygiene practices in Turkey	
4. Propositions/expressions related to the international definition of occupational hygiene as a profession	
5. Propositions/expressions related to the official recognition of practical applications for occupational hygiene in Turkey	
6. Suggestions for training standards in occupational hygiene/for occupational hygienists	
7. Expressions that describe the steps related to priorities and milestones in the development of occupational hygienists in Turkey	
8. Suggestions for competent institutions and functions for occupational hygiene/hygienists	

The second round questions was circulated to the same key contacts from the first round. In Round 2, we asked respondents about their level of agreement on the items established by the main arguments from the first round. Here, the total agreement was given as 7 points, while no agreement was given 1 point. Mean and median values were then evaluated, and a consensus frequency was determined for the group as a whole. A priori criterion of consensus was determined at 80% or greater (an agreement level of 6 or more given the point rankings). Subsections were not presented in any particular order to avoid influencing respondents. Although a section for comments was included, suggestions for additional items were not invited in this round.

Preliminary consensus results were reported in the third round. A SWOT analysis was also applied to the results from the workshop model forum, which was designed to realize a consensus on the issues determined during the researcher group sessions. On the topic of “implementation standards for occupational hygiene in Turkey”; “the existence of authorized laboratories and the relevance of the Ministry and the professional organizations to the issue” were found to be strengths, while “specialization and lack of competencies” were the main weaknesses. SWOT analyzes for “training and education standards for occupational hygiene in Turkey” showed the strength as “the society (TROHA) can work together with the Ministry”, on the other hand “challenges in requirements of multidisciplinary working” was the weakness. The 3rd group has analyzed the subject entitled “requirements and priorities for occupational hygiene in Turkey” and they expressed that “presence of the society and presence of good practicing examples” were the strengths while “absence of occupational hygiene/hygienist definition in the country” was the weakness. The last group worked on “the functionality of competent institutions in Turkey regarding occupational hygiene/hygienists”. While “effective and inveterate structure of the Ministry of Labor and Social Security and having an international relationship of the TROHA” were the strengths, the absence of the standards on education and profession in the area of occupational hygiene in Turkey was the weakness. The opportunity has been found to be having a new society about occupational hygiene such as TROHA. The main threat was changing the system and decision-makers in authorized institutions. The SWOT analysis was conducted to identify the necessary steps and institutions for the model proposal based on summary information in the highest-consensus and lowest-consensus groups. The group then used the SWOT analysis to reveal any policy impacts (this was done after the third Delphi round, in which researchers neutrally guided the group work after receiving an expert opinion that a SWOT analysis was appropriate) [39]. This was the main modification performed at the end of questionnaire-based Delphi survey, which was designed to define the roles, responsible institutions, and priorities after announcing the consensus level among items. The group then summarized its results and presented them to all participants and the report content was finally decided. This report was communicated to all related institutions and stakeholders. After receiving external validation, the report was shared with all stakeholders and international partners and posted online [29].
